# The impact of age on outcomes of breast cancer in different hormone receptor and HER2 groups

**DOI:** 10.1371/journal.pone.0280474

**Published:** 2023-01-18

**Authors:** Hongjuan Zheng, Chenyang Ge, Haiping Lin, Shishi Zhou, Wanfen Tang, Qinghua Wang, Xia Zhang, Xiayun Jin, Xifeng Xu, Jinlin Du, Jianfei Fu

**Affiliations:** 1 Department of Medical Oncology, Affiliated Jinhua Hospital, Zhejiang University School of Medicine, Jinhua, Zhejiang Province, China; 2 Department of Colorectal Surgery, Affiliated Jinhua Hospital, Zhejiang University School of Medicine, Jinhua, Zhejiang Province, China; 3 Department of Hepatobiliary Surgery, Affiliated Jinhua Hospital, Zhejiang University School of Medicine, Jinhua, Zhejiang Province, China; Nanjing Medical University, CHINA

## Abstract

**Objective:**

The aim of the current study was to explore the association between age and outcomes in breast cancer.

**Methods:**

Patients during 2010–2015 were identified from the Surveillance, Epidemiology, and End Results (SEER) database. Overall survival (OS) and breast cancer-specific death (BCSD) were taken as endpoints. The restrict cubic spline graph (RCS) was used to explore the relationship between age and outcomes in patients, and the cumulative incidence of BCSD and non-BCSD was calculated using the Gray method. Age-specific gene expression profiles were studied using RNA sequence data from the Cancer Genome Atlas (TCGA) database to explore whether there were young age-related gene or gene sets.

**Results:**

A total of 142,755 patients with breast cancer were included. The hazard ratio (HR) of OS for Patients with stage I-III breast cancer was roughly stable before 53 years old and increased significantly after that, and the HR of BCSD for these patients showed a U-shaped distribution when plotted against age, with patients younger than 50 years and patients older than 70 years experiencing the worst survival. Further stratified analysis according to molecular subtype revealed that the U-shaped distribution of the HR of BCSD with was only found in the Hormone receptor-positive/HER2-negative (HoR+/HER2-) subgroup. The cumulative incidence plots showed that young age was associated with worse BCSD in the breast cancer patients with stage I-III and HoR+/HER2- subgroup. In stage IV breast cancer, there was a linearity of the relationship between poor OS and increasing age. We failed to find any differentially expressed age-specific genes between 20–40 years and 41–60 years groups in 258 patients with stage I-III and HoR+/HER2- subtype.

**Conclusion:**

Young age could predict worse BCSD of patient with stage I-III and HoR+/HER2- breast cancer. The escalating therapy was recommended to young age breast cancer with stage I-III and HoR+/HER2- subtype.

## Introduction

In worldwide, breast cancer is the most commonly cancer in women [1], and its incidence rates is expected to increase significantly the next 5–10 years [2]. Breast cancer is undoubtedly the leading cause of cancer-related deaths in young women, especially in developing countries [3]. Traditionally, a young age at the diagnosis of breast cancer in women has been considered an independent adverse prognostic factor associated with a higher risk of relapse and death [4–8]. In 2007, St. Gallen Expert Consensus Report found young age (less than 35 years) was considered as a poor prognostic factor [9]. However, in later version of St. Gallen guideline, the age was not considered as an independent prognosis factor to make decision [10]. The same recommend also was found as for the National Comprehensive Cancer Network (NCCN) [11, 12] and European Society of Medical Oncology (EMSO) [13] guidelines. In the previous period, more studies found that young age breast cancer presented advanced stages at diagnosis [14, 15], more aggressive pathological characteristics, a greater rate of triple-negative and HER2-overexpressing tumors [16], increased risk of locoregional recurrence in young women with breast cancer compared with older ones [17]. Previous studies showed that young age breast cancer has potentially unique, aggressive and complex biological feature, with unique gene entity [6, 16, 18]. However, with an improved understanding of the molecular subtype of breast cancer, the prognostic relevance of young age in and of itself is highly controversial [19]. Whether young age is an independent prognostic marker for adverse survival even in the same molecular subtypes remains controversial [17, 20, 21]. Therefore, more guidelines no longer recommend treatment based on age alone. However, younger than 35 years of age was considered to be at higher risk for poor outcomes according to conjoint analysis for SOFT and TEXT trials [22]. Age has once again become a hot topic.

Age is an unavoidable "covariate" for all studies, including big data study, real-world study or randomized controlled trial (RCT), and researchers had to figure out how to adjust for age to minimize its impact on outcomes. Should we treat age as a continuous variable, disordered categorical variable or an ordinal categorical variable?

The differences in the prognostic relevance of young age in and of itself might be because of the various definitions of “young women”. Through previous study, the age of 40 years was defined as a reasonable cutoff value [23]. The adverse prognosis of young women only appeared in the low risk subgroup [24]. Besides, the impact of age on prognosis was related with the endpoint of studies [25], and age played different roles in different stage of breast cancer.

Restrict cubic spline (RCS) method is useful in analyzing the tendency between a continuous variable and the outcome. The dataset retrieved from the Surveillance, Epidemiology, and End Results (SEER) database is best used for trend analysis, because SEER is a nationally representative population with a large amount of data, however with some key fields missing. Therefore, big data based on SEER were analyzed by RCS method to evaluate the impact of age on the outcome.

## Method

### Study population

The SEER database is sponsored by the National Cancer Institution, which is collected and released annually. The specific methods for using SEER database were detailed in our previous research [23, 25, 26]. We identify patients diagnosed with breast cancer from January 2010 to December 2015 from SEER database. We did not include patients who diagnosed after 2015, because these patients did not have adequate follow-up time. The cutoff of 2010 was selected because from 2010 information regarding HER2 was available. We retrieved records of year and age at diagnosis, gender, race, insurance, marital status, histological type, differentiated grade, location of tumor, T-classification, N-classification, stage TNM, administration of radiotherapy, administration of chemotherapy, hormone receptor (HoR), HER2, survival months, and cause of death.

### Inclusion and exclusion criteria

The specific inclusion criteria were as follows: (1) site record ICD-O-3 was limited to breast cancer (C500-506; C508-509); (2) gender was limited to female; (3) histological type ICD-O-3 was limited to infiltrative ductal cancer (IDC), infiltrative lobar cancer (ILC) or mixed with both of them (8500/3, 8520/3, 8521/3, 8522/3, 8523/3, 8524/3); (4) the survival time of patients exceeded 1 months, (5) the age at diagnosis was limited from 20 to 80; (6) patients were not multiple primary tumors. The exclusion criteria were as follows: (1) patients were lacking documentation of race, marital status, insurance status, differentiated grade, location of tumor, HoR, HER2; (2) the cause of death was unknown; (3) patients were without surgery (For the detailed inclusion and exclusion criteria, see the **S1 Fig**).

### Variables examined

Age was considered a continuous variable when analyzing the relationship between age and outcomes of breast cancer patients. However, the patients were divided into four subgroups according to age: 20–40 years, 41–60 years, 61–70 years, and 71–80 years [26, 27] when analyzing the differences of characteristics among different age groups or taking univariate and multivariate analysis for overall survival (OS) and breast cancer-specific death (BCSD) in patients with stage I-III breast cancer. Race was divided into white, black and other. Marital status was regrouped as married, single and divorced. Insurance status was divided into insured, medicaid and uninsured. Histological type was grouped as IDC, ILC and mixture (IDC&ILC). Differentiated grade was classified as well, moderate and poor. Stage was divided into stage I, stage II, stage III and stage IV. The variable of chemotherapy was only classified as “yes” or “no/unknown”, and the variable of radiotherapy was classified as “yes” or “no”. All identified patients were divided into three groups according to hormone receptor (HoR) and HER2 status including HoR+/HER2- group, anyHoR/HER2+ group and HoR-/HER2- group.

### Statistical analysis

Chi-Squared tests was used to analyze the distribution of clinicopathological characteristic in different HoR and HER2 status groups. The OS was calculated from the date of diagnosis to the date of death. The BCSD was calculated from the date of diagnosis to the date of death of breast cancer. Alive were defined as censored, and the non-BCSD was considered a competing event. The cumulative incidence of BCSD or non-BCSD was estimated and compared via Gray’s test. The hazard ratio (HR) of OS was estimated using Cox proportional hazard regression model. The statistical method RCS was used to explore the relationship between age and outcomes of patients with breast cancer. The HR of BCSD was calculated by two steps. The first step was to generate a new dataset using the weight assignment method by time start and time stop using *crprep* function of *mstate* package. The second step was to plot RCS curve according to Cox proportional hazard model.

R3.5.3 software (http://www.r-project.org/) was used to perform the statistical analyses. And the *rms*, *cmprsk*, *mstate*, *and prodlim* package in R was used for drawing RCS plots and cumulative incidence plots. The two-sided P value less than 0.05 was considered statistically significant.

### Gene expression profile analysis

The Cancer Genome Atlas (TCGA) dataset is the largest gene database (https://gdc-portal.nci.nih.gov). For a detailed introduction to the TCGA database, see our previous research [23]. According to the following exclusion criteria, 258 patients were included. They were used to identify differentially expressed genes using the *limma* and *edgeR* packages in R. Only genes with an adjusted P value (q value) < 0.05 (Benjamini-Hochberg correction for multiple testing) were considered significant.

The specific exclusion criteria were as follows: (1) patients were lacking documentation of year and age at diagnosis, gender, T-classification, N-classification, Estrogen receptor (ER), Progesterone receptor (PR), HER2 status; (2) the age at diagnosis was younger than 20 years or older than 80 years; (3) patients were without HoR+/HER2- subtype; (4) patients has distant metastasis; (5) histological type ICD-O-3 was not IDC, ILC or mixed with both of them (8500/3, 8520/3, 8521/3, 8522/3, 8523/3, 8524/3).

## Results

### Clinicopathological characteristics of patients with stage I-III breast cancer

We identified 137,217 eligible patients with stage I-III breast cancer from SEER. The endpoint date of the follow-up was December 2015 with a median follow-up of 42 months (range: 1 to 83 months). There were 5.61% breast cancer patients aged younger than 40 years. Compared with older patients (more than 40 years), younger women (less than 40 years) were significantly associated with more IDC (92.57%), poorly differentiated tumor (51.40%), late-stage tumor (46.59% of stage II tumor; 12.65% of stage III tumor), or more HER2 over-expression and triple-negative subtype (20.86% of HER2 over-expression subtype; 15.89% of triple-negative subtype). The detail information was indicated in **Table 1**.

**Table 1:** The characteristics of patients with stage I-III breast cancer.

Risk factors	N (%)	≤40 years	41–60 years	61–70 years	71–80 years	*P* ^a^
		N (%)	N (%)	N (%)	N (%)	
**Total**	137217	7702(5.61)	65347(47.62)	40836(29.76)	23332(17.00)	
**Race**						<0.001
White	109810	5609(72.83)	51020(78.08)	33552(82.16)	19629(84.13)	
Black	13715	996(12.93)	6986(10.69)	3739(9.16)	1994(8.55)	
Others	13692	1097(14.24)	7341(11.23)	3545(8.68)	1709(7.32)	
**Marital status**						<0.001
Married	86079	4934(64.06)	43862(67.12)	25282(61.91)	12001(51.44)	
Single	20444	2149(27.90)	11221(17.17)	5012(12.27)	2062(8.84)	
Divorced	30694	619(8.04)	10264(15.71)	10542(25.82)	9269(39.73)	
**insurance**						<0.001
Insured	120956	6328(82.16)	55975(85.66)	37018(90.65)	21635(92.73)	
Medicaid	14430	1181(15.33)	8163(12.49)	3473(8.50)	1613(6.91)	
Uninsured	1831	193(2.51)	1209(1.85)	345(0.84)	84(0.36)	
**Histological type**						<0.001
IDC	116190	7130(92.57)	55888(85.52)	34011(83.29)	19161(82.12)	
ILC	12883	235(3.05)	5583(8.54)	4323(10.59)	2742(11.75)	
IDC&ILC	8144	337(4.38)	3876(5.93)	2502(6.13)	1429(6.12)	
**Differentiated grade**						<0.001
Well	33973	815(10.58)	15200(23.26)	11369(27.84)	6589(28.24)	
Moderate	62270	2928(38.02)	28897(44.22)	19235(47.10)	11210(48.05)	
Poor	40974	3959(51.40)	21250(32.52)	10232(25.06)	5533(23.71)	
**Stage**						<0.001
**Stage** I	81078	3140(40.77)	36988(56.60)	26066(63.83)	14884(63.79)	
**Stage** II	45473	3588(46.59)	22866(34.99)	12107(29.65)	6912(29.62)	
**Stage** III	10666	974(12.65)	5493(8.41)	2663(6.52)	1536(6.58)	
**HoR/HER2 status**						<0.001
HoR+/HER2-	106279	4871(63.24)	49322(75.48)	32926(80.63)	19160(82.12)	
AnyHoR/HER2+	17804	1607(20.86)	9499(14.54)	4443(10.88)	2255(9.66)	
HoR-/HER2-	13134	1224(15.89)	6526(9.99)	3467(8.49)	1917(8.22)	
**Chemotherapy**						<0.001
No/Unknown	82867	2226(28.90)	34084(52.16)	27473(67.28)	19084(81.79)	
Yes	54350	5476(71.10)	31263(47.84)	13363(32.72)	4248(18.21)	
**Radiotherapy**						<0.001
No	55018	4205(54.60)	26825(41.05)	13980(34.23)	10008(42.89)	
Yes	82199	3497(45.40)	38522(58.95)	26856(65.77)	13324(57.11)	

Abbreviations: N: number. IDC: Infiltrating duct carcinoma. ILC: Infiltrating lobular carcinoma. HoR: Hormone receptor.

^a^ P values obtained from the χ2 test. All statistical tests were two-sided.

### Impact of age on outcomes in patients with stage I-III breast cancer

#### The RCS plots about the HR of OS plotted against the age

**Fig 1:**
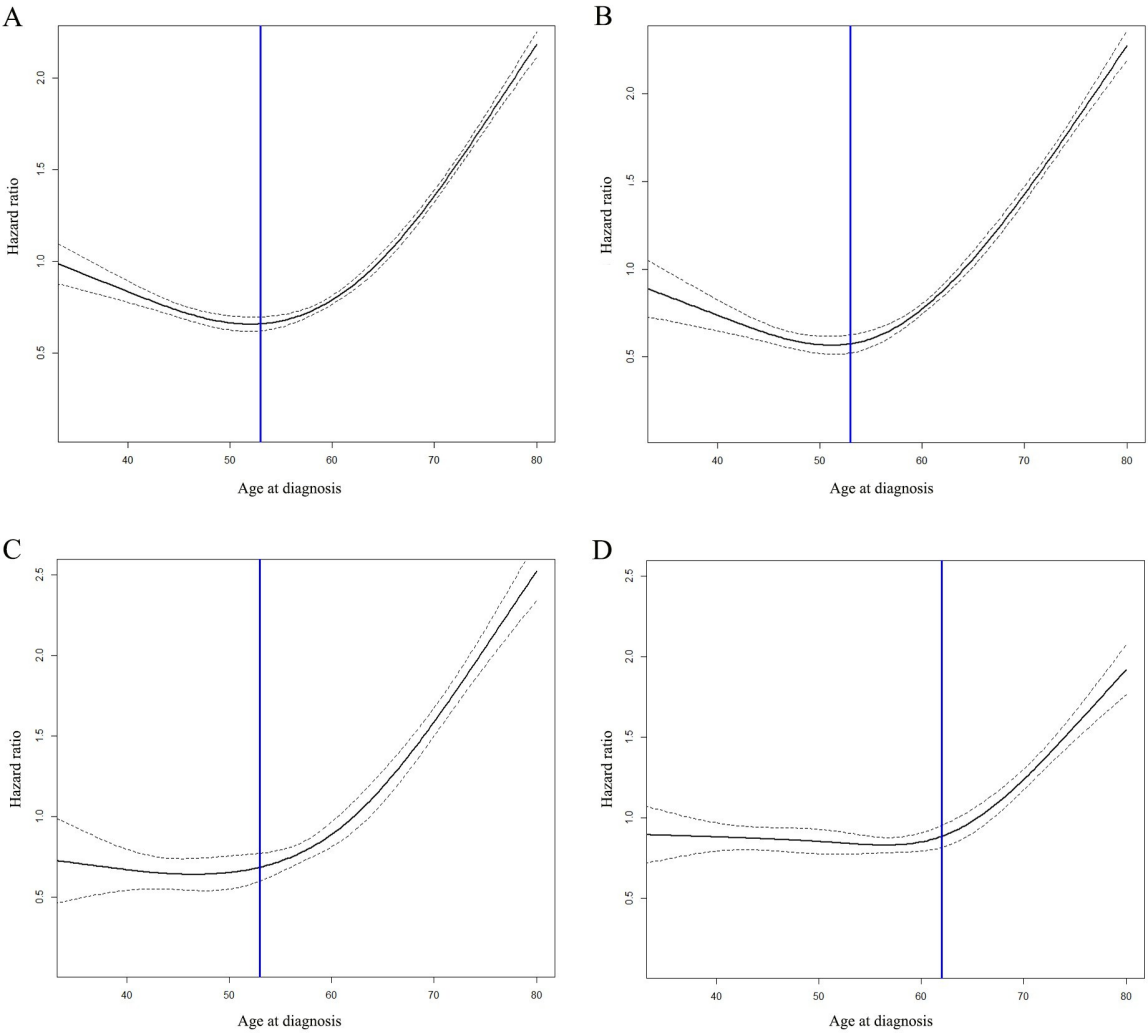
The impact of age on the overall survival (OS). The impact of age on the OS of patients with stage I-III breast cancer (A). The impact of age on OS of patients with stage I-III breast cancer in HoR+/HER2- group (B). The impact of age on OS of patients with stage I-III breast cancer in anyHoR/HER2+ group (C). The impact of age on OS of patients with stage I-III breast cancer in HoR-/HER2- group (D).

Cox proportional hazard model with continued variable of age after transformation with RCS was plotted to examine the relationship between age and the HR of OS in patients with stage I-III breast cancer (**Fig 1**). The HR of OS generally continued to stabilized before 53 years of age and then increased significantly after 53 years according to the Cox model, which could be seen in HoR+/HER2- and anyHoR/HER2+ groups. In the HoR-/HER2- group, the HR of OS began to increase significantly until 62 years of age.

#### The RCS plots about the HR of BCSD plotted against the age

**Fig 2:**
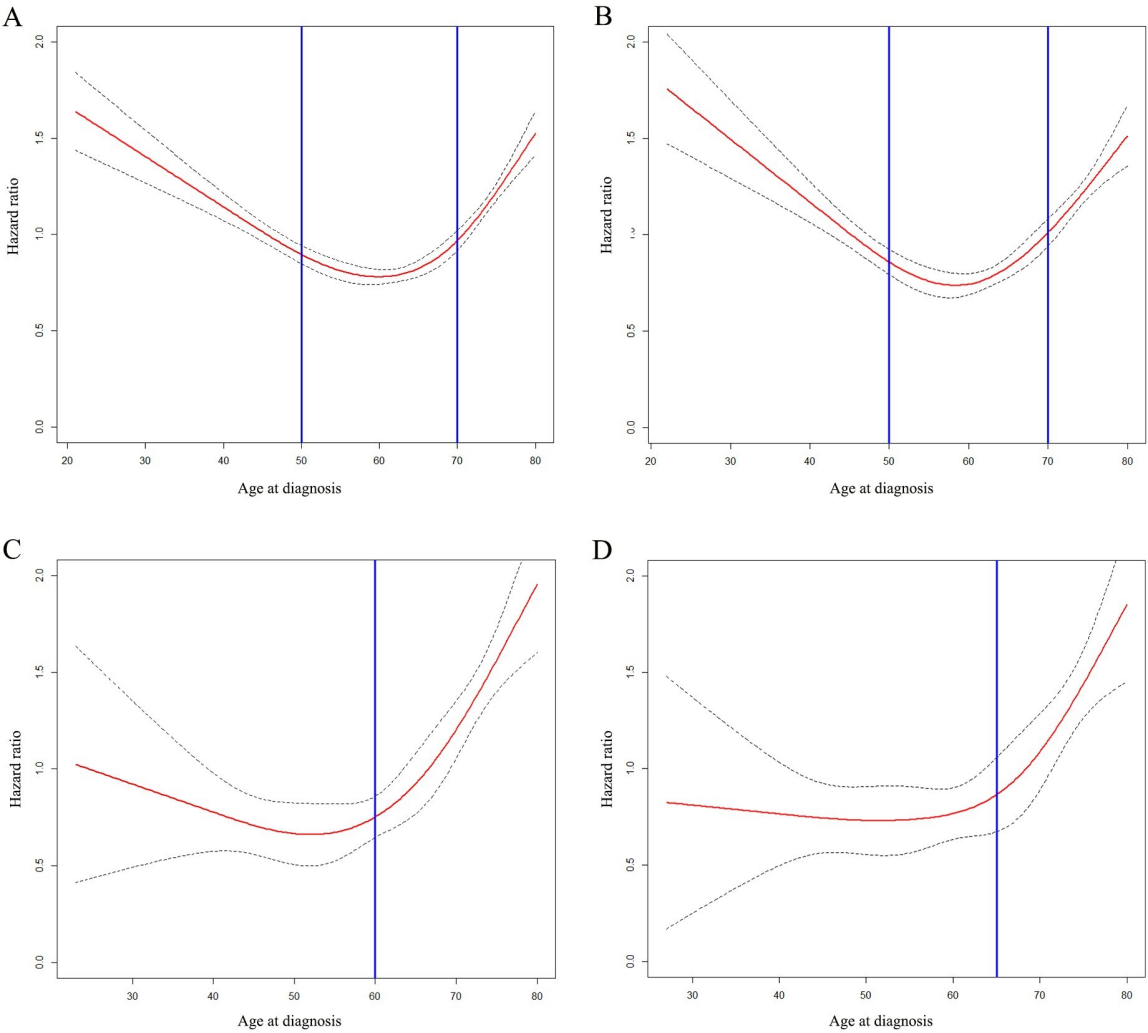
The impact of age on the breast cancer-specific death (BCSD). The impact of age on the BCSD of patients with stage I-III breast cancer (A). The impact of age on BCSD of patients with stage I-III breast cancer in HoR+/HER2- group (B). The impact of age on BCSD of patients with stage I-III breast cancer in anyHoR/HER2+ group (C). The impact of age on BCSD of patients with stage I-III breast cancer in HoR-/HER2- group (D).

In patients with stage I-III breast cancer, the overall analysis showed that when plotted against the age, the HR of BCSD was a U-shaped curve, which was also observed in the HoR+/HER2- group. However, in the anyHoR/HER2+ group, the HR remained relatively steady before 60 years old and increased rapidly after that. In the HoR-/HER2- group, the HR continued to stabilized before 65 years and increase slowly after that. The detailed information is indicated in **Fig 2**.

#### The RCS plots about the HR of BCSD plotted against the age in different therapy status

We further explored patients with stage I-III breast cancer according to different therapy status and the results showed that the U-shaped curve was only observed in the therapy cohort (patients received chemotherapy or radiotherapy). The detailed information is indicated in **S2 Fig**.

**Fig 3:**
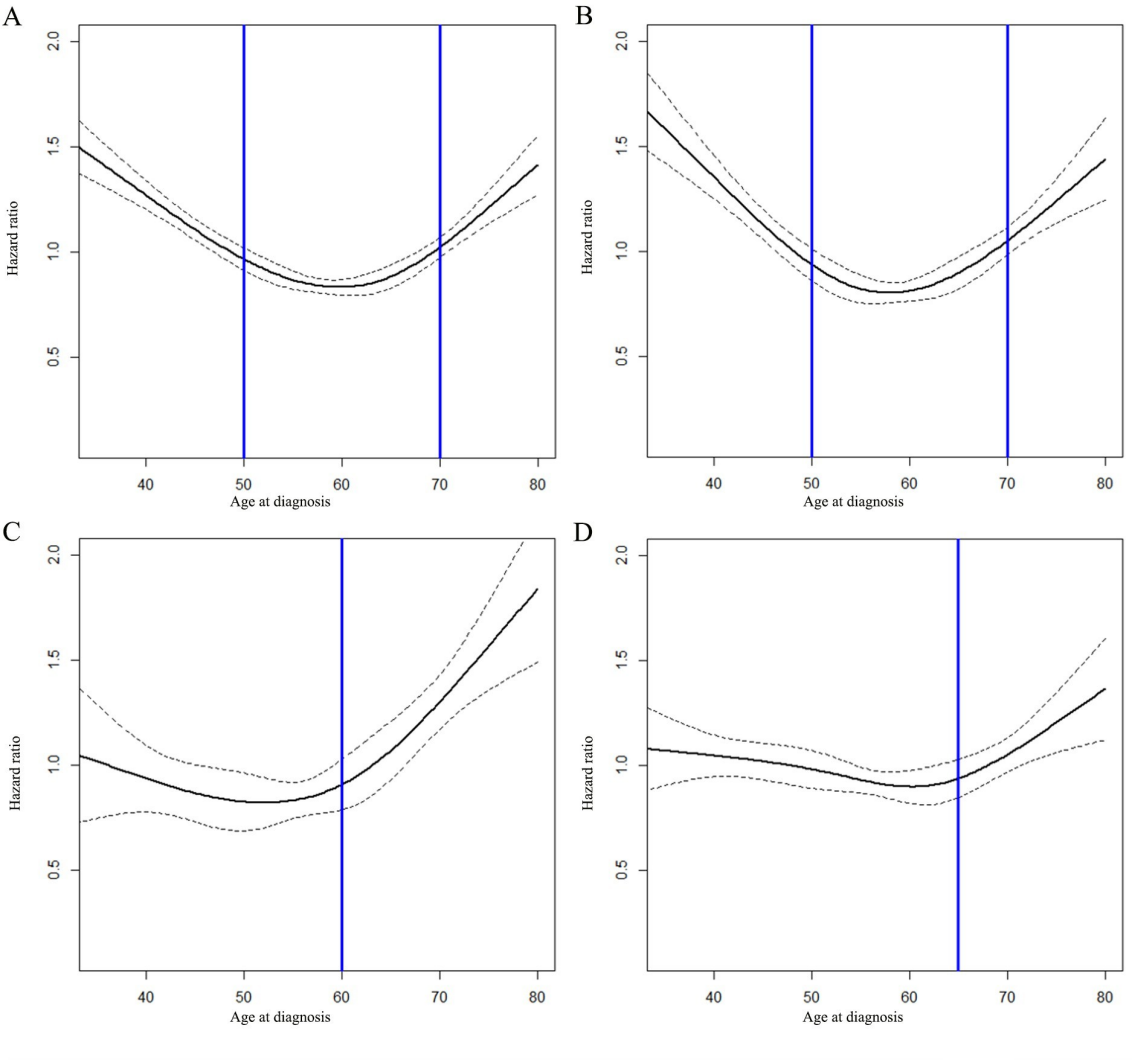
The impact of age on the breast cancer-specific death (BCSD) of patients with stage I-III breast cancer in therapy cohort. The impact of age on the BCSD of patients with stage I-III breast cancer (A). The impact of age on BCSD of patients with stage I-III breast cancer in HoR+/HER2- group (B). The impact of age on BCSD of patients with stage I-III breast cancer in anyHoR/HER2+ group (C). The impact of age on BCSD of patients with stage I-III breast cancer in HoR-/HER2- group (D).

In the therapy cohort, we further analyzed the HR of BCSD according to the molecular subtypes of the patients, and the results showed that the HR of BCSD was a U-shaped curve in the HoR+/HER2- group (**Fig 3**). Most young patients received chemotherapy or radiotherapy (**Table 1**), resulting in the **impact of age on BCSD in the therapy cohort** similar to that in **Fig 2**.

#### The cumulative incidence plots in patients with stage I-III breast cancer

**Fig 4:**
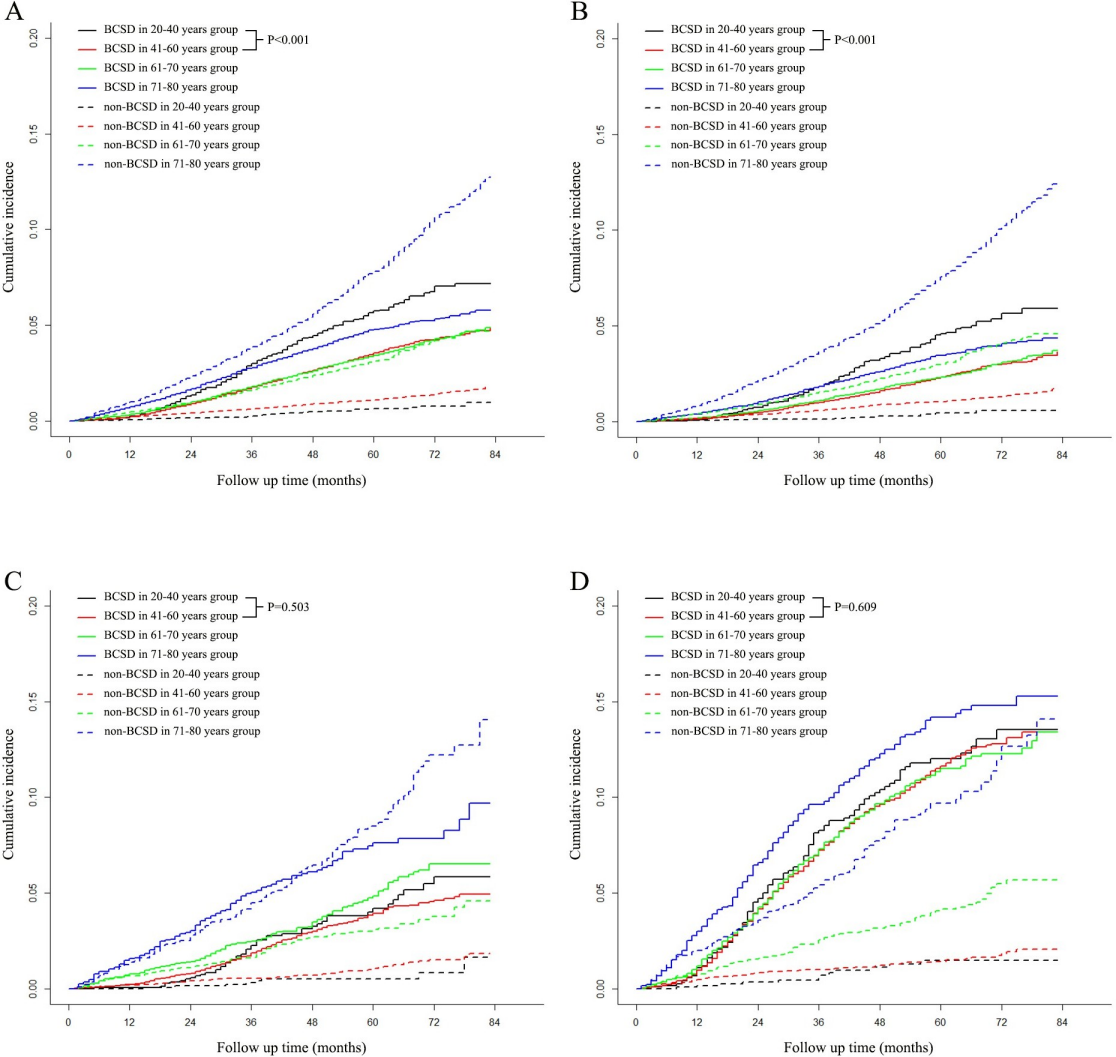
Cumulative incidence of the breast cancer-specific death (BCSD) and non-BCSD for patients with stage I-III breast cancer. In all patients or the patients with HoR+/HER2- subtype, 20–40 years old group had more BCSD compared to 41–60 years group with p < 0.001 (A, B). However, in the patients with anyHoR/HER2+ subtype or in HoR-/HER2-subtype, 20–40 years old group had similar BCSD compared to 41–60 years group with P equal to 0.503 and 0.609, respectively (C, D). The curves were plotted using the Gray method.

The cumulative incidence plots according to Gray method showed that in the breast cancer patients with stage I-III or with stage I-III and HoR+/HER2- subtype, patients with young age (less than 40 years) had more BCSD compared to the patients with 41–60 years (both with *P* < 0.001). Whereas, in the patients with anyHoR/HER2+ or HoR-/HER2- subtypes, there was no statistically significant difference in BCSD between young age group and 41–60 years group (with *P* equal to 0.503 and 0.609, respectively). The detailed information is indicated in **Fig 4**.

### Impact of age on outcomes in patients with stage IV breast cancer

**Fig 5:**
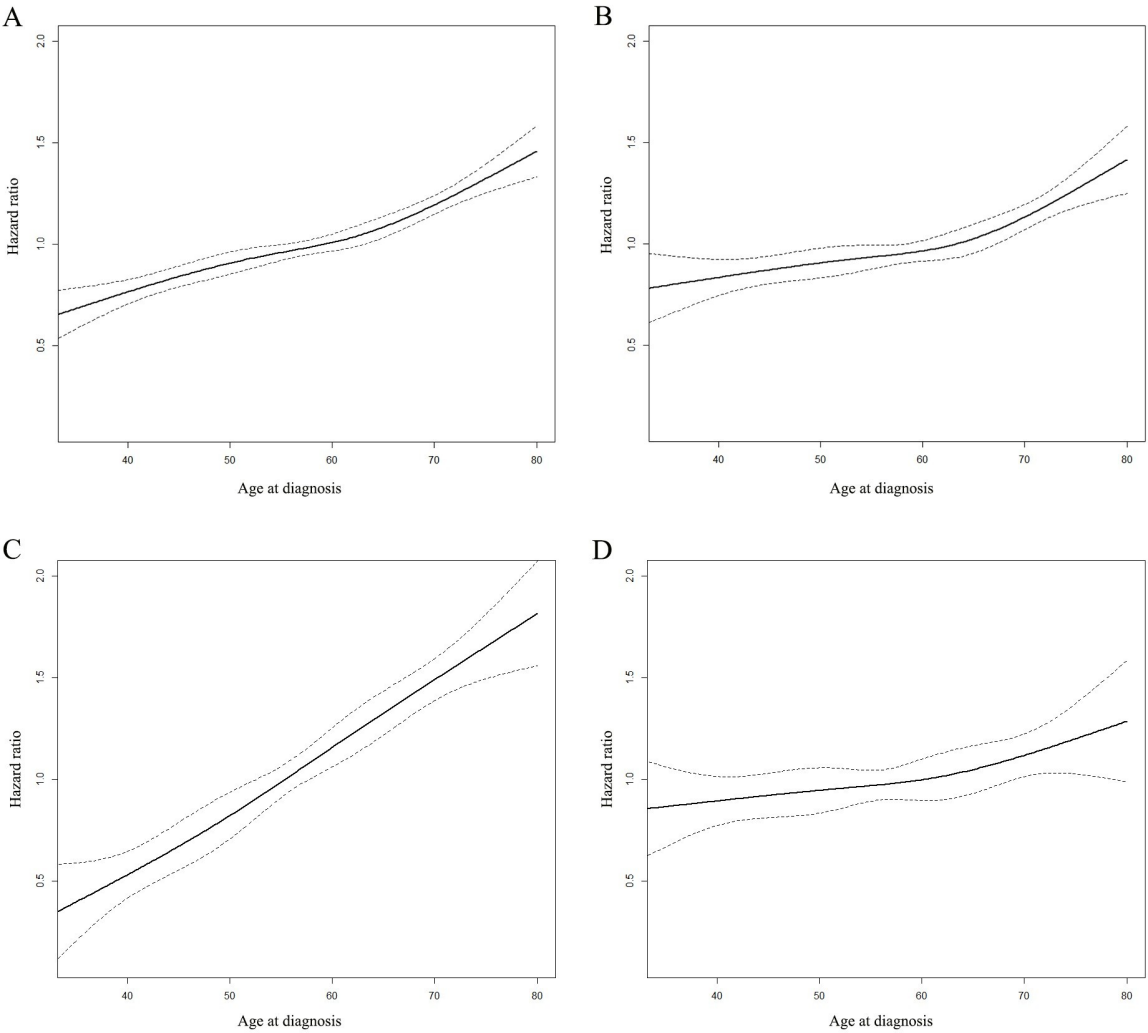
The impact of age on the overall survival (OS). The impact of age on the OS of patients with stage IV breast cancer (A). The impact of age on OS of patients with stage IV breast cancer in HoR+/HER2- group (B). The impact of age on OS of patients with stage IV breast cancer in anyHoR/HER2+ group (C). The impact of age on OS of patients with stage IV breast cancer in HoR-/HER2- group (D).

We identified 5,538 eligible patients with stage IV breast cancer from SEER for further analysis, and the detail clinicopathological characteristics of them was indicated in **S1 Table**. Regardless of HoR and HER2 status, there was a linearity of the relationship between the HR of OS and age in stage IV breast cancer. The HR of OS gradually increased with age (**Fig 5**). The relationship between the HR of BCSD and age was not further analyzed, due to the low ratio of competing event in the patients with stage IV breast cancer.

### Gene expression profile analysis in breast cancer patients with stage I-III and HoR+/HER2- subtype

In breast cancer patients with stage I-III and HoR+/HER2- subtype, the HR of BCSD showed a U-shaped distribution, and young age was associated with worse BCSD. Therefore, we identified 258 breast cancer patients with stage I-III and HoR+/HER2- subtype from TCGA dataset to compare the differentially expressed age-related genes between 20–40 years and 41–60 years groups. There were 42 patients in the 20–40 years group and 216 patients in the 41–60 years group. The detail characteristics of these patients were indicated in **Table 2**.

**Table 2:** The characteristics of 258 patients with stage I-III and HoR+/HER2- breast cancer in TCGA.

Characteristic	Number (%)	20–40 years	41–60 years	*P* ^a^
**Total**	258	42(16.28)	216(83.72)	
**Race**				0.018
White	201	28(66.67)	173(80.09)	
Black	23	7(16.67)	16(7.41)	
Others	17	6(14.29)	11(5.09)	
Unknown	17	1(2.38)	16(7.41)	
**Location**				0.578
Upper-outer	119	15(35.71)	104(48.15)	
Lower-outer	35	6(14.29)	29(13.43)	
Upper-inner	39	7(16.67)	32(14.81)	
Lower-inner	7	2(4.76)	5(2.31)	
Overlap	58	12(28.57)	46(21.3)	
**Histological type**				0.090
IDC	208	39(92.86)	169(78.24)	
ILC	36	2(4.76)	34(15.74)	
IDC and ILC	14	1(2.38)	13(6.02)	
**T-Classification** ^b^				0.716
T1	72	12(28.57)	60(27.78)	
T2	149	26(61.9)	123(56.94)	
T3	33	4(9.52)	29(13.43)	
T4	4	0(0)	4(1.85)	
**N-Classification** ^b^				0.263
N0	106	14(33.33)	92(42.59)	
N1	103	18(42.86)	85(39.35)	
N2	35	9(21.43)	26(12.04)	
N3	14	1(2.38)	13(6.02)	
**Stage** ^b^				0.570
Stage I	46	9(21.43)	37(17.13)	
Stage II	148	21(50)	127(58.8)	
Stage III	64	12(28.57)	52(24.07)	
**Primary Surgery**				0.948
Lumpectomy	52	10(23.81)	42(19.44)	
MRS	72	10(23.81)	62(28.7)	
Mastectomy	51	9(21.43)	42(19.44)	
Other	70	11(26.19)	59(27.31)	
Unknown	13	2(4.76)	11(5.09)	
**Chemotherapy**				1.000
Yes	172	28(66.67)	144(66.67)	
No	86	14(33.33)	72(33.33)	
**Endocrine therapy**				0.807
Yes	164	26(61.9)	138(63.89)	
No	94	16(38.1)	78(36.11)	
**Targeted therapy**				0.442
Yes	3	0(0)	3(1.39)	
No	255	42(100)	213(98.61)	
**Radiotherapy**				0.734
Yes	127	19(45.24)	108(50)	
No	86	14(33.33)	72(33.33)	
Unknown	45	9(21.43)	36(16.67)	
**ER**				0.634
Negative	4	1(2.38)	3(1.39)	
Positive	254	41(97.62)	213(98.61)	
**PR**				0.521
Negative	35	7(16.67)	28(12.96)	
Positive	223	35(83.33)	188(87.04)	
**Subtype (PAM50)**				0.303
Luminal A	156	31(73.81)	125(57.87)	
Luminal B	70	9(21.43)	61(28.24)	
HER2	0	0	0	
Basal	6	0(0)	6(2.78)	
Normal	8	1(2.38)	7(3.24)	
Unknown	18	1(2.38)	17(7.87)	

Abbreviations: TGCA: The Cancer Genome Atlas. IDC: Infiltrating duct carcinoma. ILC: Infiltrating lobular carcinoma. HoR: Hormone receptor. MRS: Modified Radical Mastectomy. ER: Estrogen receptor. PR: progesterone receptor. PAM50: 50-gene prediction analysis of a microarray.

^a^ P values obtained from the χ2 test. All statistical tests were two-sided.

^b^ T and N stage are according to the 7th edition of the American Joint Committee on Cancer (AJCC) staging system.

There were 20,530 age-related genes in the IlluminaHiSeq RNASeqV2 platforms of the TCGA dataset. After filtering through the CPM protocol and standardizing by the TMM method, the 19961 genes were eventually included in the subsequent analysis. Finally, there were no differentially expressed genes between 20–40 years and 41–60 years groups after fitting by the negative binomial generalized log-linear model, and proofreading by FDR.

## Discussion

It is definitely that the prognosis of old patients with breast cancer is worse, whether the endpoint is OS or BCSD, which is consistent with the results of our study. However, young age has different impact on outcomes. Therefore, young age is the key factor whether age is an independent prognostic marker for outcomes in patients with breast cancer. Some studies suggested young age of breast cancer to be an independent prognostic factor of adverse outcome [4–9]. However, a few studies revealed that age was not an independent prognosis factor [10]. The reason for these two opposite conclusions was that they did not take into account the stage and molecular subtypes of breast cancer. In this study, the effect of age on the prognosis of breast cancer was explored on the basis of full combination of stage and molecular subtypes. Then the results showed that the U-shaped distribution of HR of BCSD exists only in the patients with stage I-III and HoR+/HER2- breast cancer. And in the patients with stage I-III and HoR+/HER2- subtype, younger than 40 years had more BCSD compared to those older than 40 years. Further explore whether there were age-related gene or gene sets in stage I-III and HoR+/HER2- breast cancer, and the results showed that there were no differentially expressed genes between 20–40 years and 41–60 years groups, which suggested that young age breast cancer is not a unique biological entity.

Why is there always a controversy about the prognostic relevance of young age in and of itself? In some previous studies, researchers always focus on the variable of age, such as the cutoff value of age, age grouping and age control, rather than the relationship between age and endpoint selection [7, 28–30]. In the study, we found that the impact of age on outcomes was completely different when setting different endpoint, OS and BCSD. The HR of OS continued to stabilized at first and then increased significantly, or liner increased completely. Young age was not a prognostic factor of worse OS, whereas, a protective factor. Probably because OS was largely impaired by non-BCSD, especially in old age patients [25]. The U-shaped distribution of the HR on outcomes has always been accepted by most scholars [31, 32]. For the first time, we propose that U-shaped distribution only existed when BCSD is defined as endpoint.

The endpoint of BCSD, which had taken into consideration, could better reflect the biological behavior of breast cancer. Through subgroup analysis, we found that the U-shaped distribution of HR merely occurred in patients with stage I-III breast cancer and HoR+/HER2- subtype, which was not found previously. This finding provides a good reference for future research and adjustment of age. In future studies concerning stage I-III and HoR+/HER2- breast cancer, we suggested that the age of 40 and 60 years are reasonable cutoff values to group the age in pre-menopausal patients diagnosed with breast cancer, and the age of 70 years are reasonable cutoff value to group the age in post-menopausal patients. Besides, the escalating therapy was recommended to the young patients (less than 40 year) with stage I-III and HoR+/HER2- breast cancer due to worse survival, which was consistent with the previous studies [17, 20, 21, 33]. In Francis’s study, endocrine therapy combined with ovarian function suppression (OFS) and escalating chemotherapy were recommended in premenopausal breast cancer [22]. In future researches about HER2-overexpressing breast cancer, old age rather than young age should be brought into focus. However, in triple-negative breast cancers, the impact of age on outcomes is minimal, and it is not necessary to divide age into too many age subgroups.

Therefore, it is best to explore the impact of young age on outcomes in patients with stage I-III and HoR+/HER2- breast cancer. The main emphasis of future research would be to explore age-related gene or gene sets through analyzing gene expression profiles of stage I-III and HoR+/HER2- breast cancer. Anders’s study [19] showed that there was no age-related gene or gene sets in young age (less than 40 years) and old age (more than 60 years) breast cancer. Older than 60 years was used as a control group in Anders’s study, which, however, was unreasonable according to our study, because the HR of BCSD exhibited U-shaped distribution. Through analyzing 3,522 patients who identified from 20 GSE datasets, Azim’s study [18] suggested that there were 7 genes and 5 gene sets associated with age, after adjustment for molecular subtype and clinicopathological characteristics. However, their study treated age as a continuous variable, which was unreasonable. Besides, Azim and co-authors further evaluated the association between age and genomic aberrations in patients identified from TCGA dataset [34]. The results showed that there were indeed age-related somatic mutations, chromosomal copy number variations (CNVs) and transcriptomic profiles. But the age grouping in this study is not reasonable, and the results were not adjusted for molecular subtype. In our previous study [23], the age of 40 years was a reasonable cutoff value for defining “young age”, and we failed to find any age-related gene in TCGA dataset. There is no evidence that young age breast cancer with HoR+/HER2- is a unique biological entity up to now.

Our study has several potential limitations. The molecular subtypes in our study were classified by a three-gene classifier rather than 50-gene prediction analysis of a microarray (PAM50) classifier. Due to retrospective analyses according to SEER database without recurrence data, we can only analyze death-related endpoints, such OS and BCSD, rather than relapse-free survival (RFS).

## Conclusion

If BCSD is taken as the endpoint, U-shaped distribution of BCSD only existed in patients with stage I-III and HoR+/HER2- breast cancer rather than the whole. We have to set age as a category variable containing four age subgroups. However, young age breast cancer is still not a unique biological entity.

## Supporting information

S1 Data (XLSX)

S1 Fig The flow chart of inclusion and exclusion criteria. (TIF)

S2 Fig The impact of age on the breast cancer-specific death (BCSD) of patients with stage I-III breast cancer in different therapy states. The impact of age on the BCSD of patients with therapy (A). The impact of age on BCSD of patients without therapy (B).
(TIF)

S1 Table The characteristics of patients with stage IV breast cancer. (DOCX)
