# Supplementary figures and images for: The impact of age on outcomes of breast cancer in different hormone receptor and HER2 groups
Source: PLoS One. 2023 Jan 18;18(1):e0280474. doi: 10.1371/journal.pone.0280474 (PMC9847906; doi:10.1371/journal.pone.0280474)

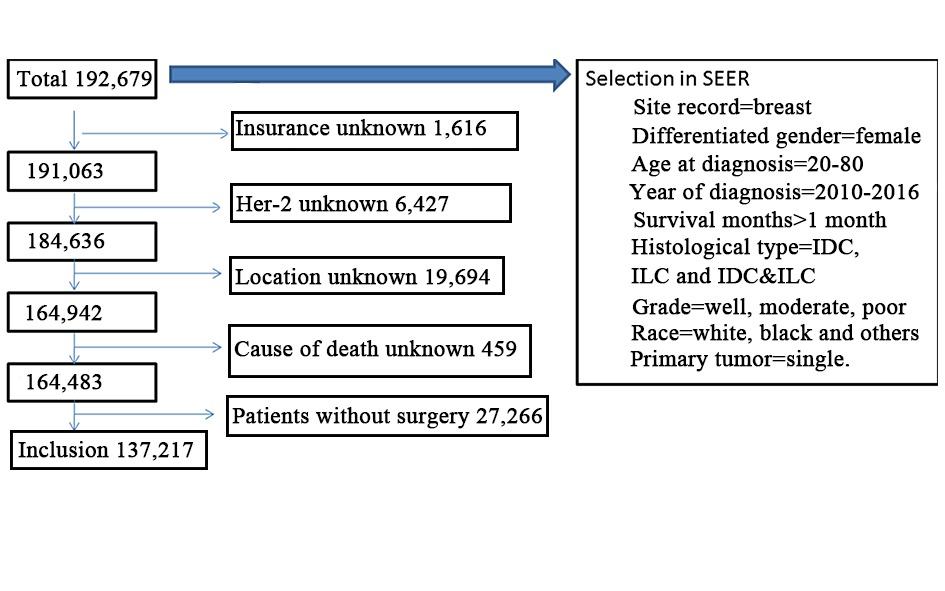

Supplement: S1 Fig — (TIF) [file pone.0280474.s002.tif]

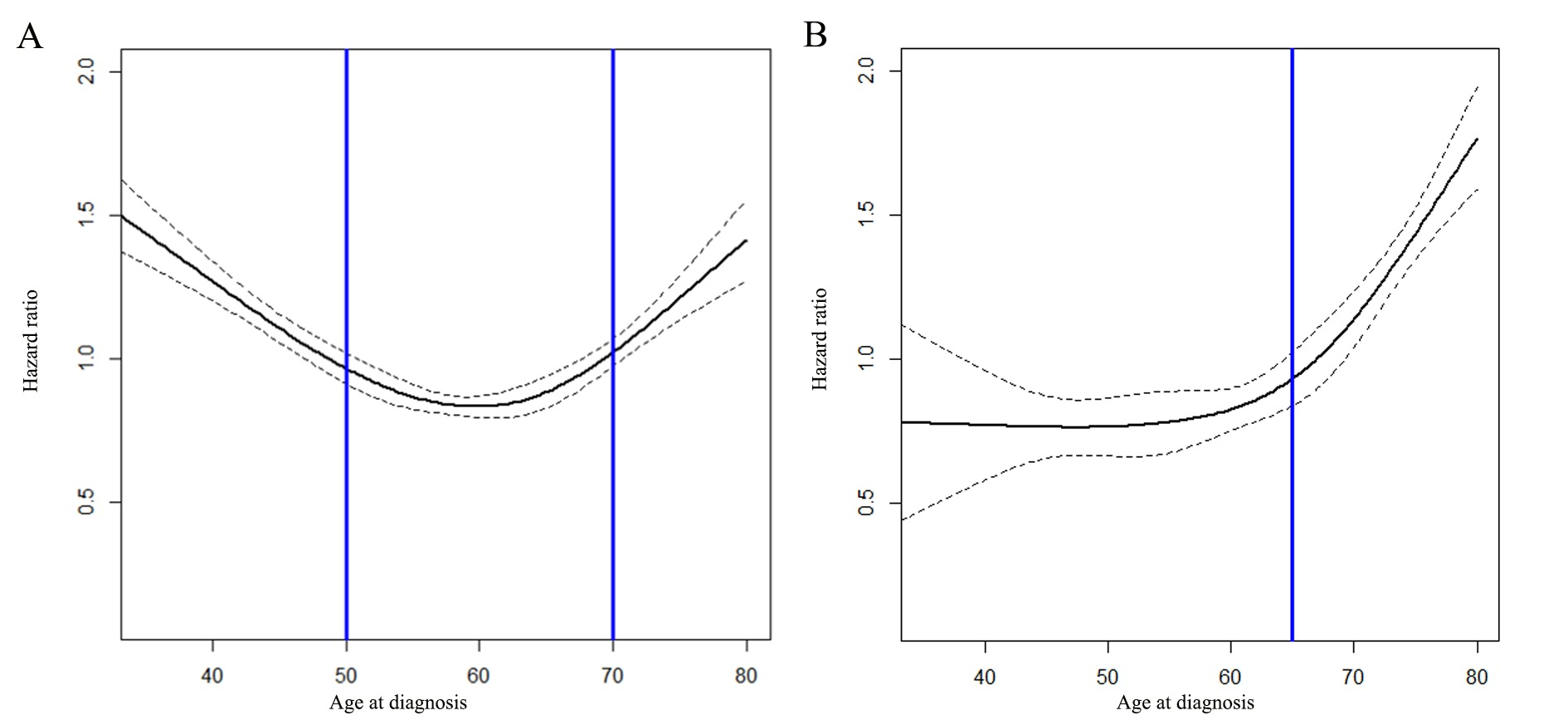

Supplement: S2 Fig — The impact of age on the BCSD of patients with therapy (A). The impact of age on BCSD of patients without therapy (B). (TIF) [file pone.0280474.s003.tif]
