# Supplementary material for: The impact of age on outcomes of breast cancer in different hormone receptor and HER2 groups
Source: PLoS One. 2023 Jan 18;18(1):e0280474. doi: 10.1371/journal.pone.0280474 (PMC9847906; doi:10.1371/journal.pone.0280474)
Supplement: S1 Table — (DOCX) [file pone.0280474.s004.docx]

**Supplemental Table 1 The characteristics of patients with stage IV breast cancer**

| **Risk factors** | N (%) | ≤40 years  N (%) | 41-60 years  N (%) | 61-70 years  N (%) | 71-80 years  N (%) | *P^a^* |
| --- | --- | --- | --- | --- | --- | --- |
| **Total** | 5538 | 608(10.98) | 2660(48.03) | 1432(25.86) | 838(15.13) |  |
| **Race** |  |  |  |  |  | <0.001 |
| White | 4158 | 410(67.43) | 1924(72.33) | 1132(79.05) | 692(82.58) |  |
| Black | 927 | 137(22.53) | 485(18.23) | 200(13.97) | 105(12.53) |  |
| Others | 453 | 61(10.03) | 251(9.44) | 100(6.98) | 41(4.89) |  |
| **Marital status** |  |  |  |  |  | <0.001 |
| Married | 2779 | 329(54.11) | 1399(52.59) | 701(48.95) | 350(41.77) |  |
| Single | 1323 | 234(38.49) | 716(26.92) | 280(19.55) | 93(11.10) |  |
| Divorced | 1436 | 45(7.40) | 545(20.49) | 451(31.49) | 395(47.14) |  |
| **insurance** |  |  |  |  |  | <0.001 |
| Insured | 4078 | 406(66.78) | 1801(67.71) | 1137(79.40) | 734(87.59) |  |
| Medicaid | 1228 | 178(29.28) | 711(26.73) | 242(16.90) | 97(11.58) |  |
| Uninsured | 232 | 24(3.95) | 148(5.56) | 53(3.70) | 7(0.84) |  |
| **Histological type** |  |  |  |  |  | <0.001 |
| IDC | 4731 | 566(93.09) | 2314(86.99) | 1167(81.49) | 684(81.62) |  |
| ILC | 515 | 15(2.47) | 214(8.05) | 179(12.50) | 107(12.77) |  |
| IDC&ILC | 292 | 27(4.44) | 132(4.96) | 86(6.01) | 47(5.61) |  |
| **Differentiated grade** |  |  |  |  |  | <0.001 |
| Well | 417 | 19(3.13) | 179(6.73) | 133(9.29) | 86(10.26) |  |
| Moderate | 2313 | 207(34.05) | 1068(40.15) | 643(44.90) | 395(47.14) |  |
| Poor | 2808 | 382(62.83) | 1413(53.12) | 656(45.81) | 357(42.6) |  |
| **HoR/HER2 status** |  |  |  |  |  | <0.001 |
| HoR+/HER2- | 3172 | 279(45.89) | 1468(55.19) | 881(61.52) | 544(64.92) |  |
| AnyHoR/HER2+ | 1583 | 239(39.31) | 789(29.66) | 369(25.77) | 186(22.20) |  |
| HoR-/HER2- | 783 | 90(14.80) | 403(15.15) | 182(12.71) | 108(12.89) |  |
| **Chemotherapy** |  |  |  |  |  | <0.001 |
| No/Unknown | 1905 | 94(15.46) | 744(27.97) | 571(39.87) | 496(59.19) |  |
| Yes | 3633 | 514(84.54) | 1916(72.03) | 861(60.13) | 342(40.81) |  |
| **Radiotherapy** |  |  |  |  |  | <0.001 |
| No | 4260 | 422(69.41) | 1992(74.89) | 1133(79.12) | 713(85.08) |  |
| Yes | 1278 | 186(30.59) | 668(25.11) | 299(20.88) | 125(14.92) |  |

Abbreviations: N: number. IDC: Infiltrating duct carcinoma. ILC: Infiltrating lobular carcinoma. HoR: Hormone receptor.

^a^ P values obtained from the χ2 test. All statistical tests were two-sided.
